# Differential expression and modulation of EBI2 and 7α,25-OHC synthesizing (CH25H, CYP7B1) and degrading (HSD3B7) enzymes in mouse and human brain vascular cells

**DOI:** 10.1371/journal.pone.0318822

**Published:** 2025-02-25

**Authors:** Fionä Caratis, Bartosz Karaszewski, Ilona Klejbor, Tomomi Furihata, Aleksandra Rutkowska

**Affiliations:** 1 Department of Anatomy and Neurobiology, Medical University of Gdansk, Gdansk, Poland; 2 Department of Adult Neurology, Medical University of Gdansk & University Clinical Center, Gdansk, Poland; 3 Brain Diseases Centre, Medical University of Gdansk, Gdansk, Poland; 4 Department of Anatomy, Collegium Medicum, Jan Kochanowski University in Kielce, Kielce, Poland; 5 Laboratory of Clinical Pharmacy and Experimental Therapeutics, School of Pharmacy, Tokyo University of Pharmacy and Life Sciences, Hachioji, Tokyo, Japan; University of Illinois at Chicago, UNITED STATES OF AMERICA

## Abstract

The endogenous ligand for the EBI2 receptor, oxysterol 7α,25OHC, crucial for immune responses, is finely regulated by CH25H, CYP7B1 and HSD3B7 enzymes. Lymphoid stromal cells and follicular dendritic cells within T cell follicles maintain a gradient of 7α,25OHC, with stromal cells increasing and dendritic cells decreasing its concentration. This gradient is pivotal for proper B cell positioning in lymphoid tissue. In the animal model of multiple sclerosis, the experimental autoimmune encephalomyelitis, the levels of 7α,25OHC rapidly increase in the central nervous system driving the migration of EBI2 expressing immune cells through the blood-brain barrier (BBB). To explore if blood vessel cells in the brain express these enzymes, we examined normal mouse brain microvessels and studied changes in their expression during inflammation. *Ebi2* was abundantly expressed in endothelial cells, pericytes/smooth muscle cells, and astrocytic endfeet. *Ch25h, Cyp7b1,* and *Hsd3b7* were variably detected in each cell type, suggesting their active involvement in oxysterol 7α,25OHC synthesis and gradient maintenance under normal conditions. Significant species-specific differences emerged in *EBI2* and the enzyme levels between mouse and human BBB-forming cells. Under acute inflammatory conditions, *Ebi2* and synthesizing enzyme modulation occurred in the brain, with the magnitude and direction of change based on the enzyme. Lastly, in an *in vitro* astrocyte migration model, CYP7B1 inhibitor clotrimazole, as well as EBI2 antagonist, NIBR189, inhibited lipopolysaccharide-induced cell migration indicating the involvement of EBI2 and its ligand in brain cell migration under inflammatory conditions.

## Introduction

The GPR183 (also known as Epstein-Barr virus-induced gene 2 (EBI2)) is a G protein-coupled receptor discovered in 1993 in EBV-infected B lymphocytes [1]. Since then, EBI2 was shown to play important roles in the regulation of the innate and adaptive immune systems, cellular migration [2–7], inflammatory signalling [8–10] and myelination in the central nervous system (CNS) [11–15]. Its most potent endogenous agonist is oxysterol 7α,25-dihydroxycholesterol (7α,25OHC) [4,16]. 7α,25OHC is synthesized from cholesterol by the sequential enzymatic activity of cholesterol 25-hydroxylase (CH25H) and 25-hydroxycholesterol 7-alpha-hydroxylase (CYP7B1) and metabolized by 3beta-hydroxy-delta(5)-C27-steroid oxidoreductase (HSD3B7) [17]. 7α,25OHC was repeatedly shown to direct immune cell migration via an oxysterol gradient, both *in vivo* and *in vitro*, in EBI2-expressing cells demonstrating its key regulatory role in immune cell function and immunity [2,4,9,18,19].

In CNS, the entry of immune cells is restricted by the blood-brain barrier (BBB), a physical barrier formed by tight junction proteins at the level of the brain microvascular endothelial cells (ECs), and further supported and regulated by pericytes, astrocytic endfeet, and other cells [20]. The function of the barrier is compromised by a range of factors including inflammation, neurodegeneration, stroke and brain trauma, to name just a few. In multiple sclerosis (MS), a chronic inflammatory disease of the CNS, disruption of the BBB occurs focally and at very early stages facilitating the entry of peripherally activated lymphocytes into the brain parenchyma. In the animal model of MS, the experimental autoimmune encephalomyelitis (EAE), increased concentration of 7α,25OHC in mouse CNS was independently reported by two groups [8,21]. Wanke and colleagues [8] established that the increase in 7α,25OHC levels resulted from the upregulation of CH25H by microglia and CYP7B1 by infiltrating lymphocytes during simultaneous downregulation of HSD3B7. Importantly, the increased concentration of the EBI2 ligand enhanced the migration of autoreactive T cells into the CNS during the early phases of transfer EAE, exacerbating the disease course. Similar conclusions were drawn in a study using the EAE model and CH25H knock-out mice [22]. The EAE severity was significantly attenuated in the CH25H deficient mice in part by reduced trafficking of encephalitogenic CD4 + T cells into the CNS. In a more recent study, the same group demonstrated that knock-out of CH25H specifically in ECs attenuates the course of EAE [23].

The levels of oxysterols in the plasma and the cerebrospinal fluid (CSF) also change dynamically in humans in the course of inflammatory diseases. Intravenous injection of lipopolysaccharide (LPS) in humans increased 25-hydroxycholesterol (25OHC) plasma concentration [24]. In another study, stimulation with a vasoactive peptide hormone angiotensin II upregulated CH25H 50-fold in cultured rat vascular smooth muscle cells [25]. Crick and colleagues (2017) measured a wide range of oxysterols in the CSF and plasma of patients diagnosed with relapsing-remitting MS, clinically isolated syndrome, neurodegenerative diseases including Parkinson’s and Alzheimer’s disease, amyotrophic lateral sclerosis and other inflammatory CNS diseases. Specifically in MS patients, the levels of 25OHC, the precursor to 7α,25OHC, were reduced in the plasma and increased only in the CSF in relapsing-remitting MS indicating disease-specific modulation of oxysterol levels in the CNS [26].

Apart from the recent identification of CH25H as a synthesizing enzyme by CNS ECs [23], the expression of EBI2 and the specific enzymes involved in the synthesis (CH25H, CYP7B1) and degradation (HSD3B7) of 7α,25OHC in the brain microvascular cells have not been investigated. Here, we examine whether EBI2 and the enzymes in the 7α,25OHC synthetic pathway (*CH25H, CYP7B1, HSD3B7*) are expressed in the brain vascular cells and if systemic inflammation modulates their levels in the brain. Regulation of 7α,25OHC levels in the brain may help in the identification of novel drug targets for the modulation of neuroinflammatory signalling and immune cell trafficking into the CNS.

## Materials and methods

### Animals

All animal experiments were approved by the Local Ethical Committee for Animal Experiments in Bydgoszcz, Poland under licence numbers 27/2019 and 38/2021. The C57BL/6 male mice were housed in standard cages with an enriched environment under 12-hour day and night cycles. The air in the room was exchanged 15 times per hour, the temperature was kept at 20–23^o^C and the humidity was between 50–60%. The animals had unrestricted access to food and water.

### LPS *in vivo* model

Forty-eight three-month-old male C57BL/6 mice were used. The mice received intraperitoneal (i.p.) injections of 0.9% NaCl (vehicle) or 2 mg/kg LPS for 12 or 24 hours (h). The mice were anaesthetised with isoflurane and perfused with NaCl for subsequent biochemical analysis or 2% paraformaldehyde (PFA) for immunohistochemistry (IHC). Whole brains were removed, snap-frozen and stored at ‒80°C for subsequent biochemical analysis or put into the O.C.T. compound and cut into 40 μm sections with a cryostat. The cut slices were submerged in anti-freeze and stored at ‒20°C until used for IHC. The snap-frozen brains (right hemispheres) were homogenised with a pestle and a mortar in liquid nitrogen. The resulting powder was then suspended in 400 μL of fenozol/50 mg of tissue and frozen at ‒80°C until needed. The left hemispheres were homogenised with a Dounce homogeniser in RIPA buffer supplemented with a protein inhibitor cocktail (700 µl/100 mg of tissue) and centrifuged at 13.4 k rpm for 20 minutes at 4°C. The supernatant was then aliquoted for ELISAs and stored at ‒80°C until needed. The pellet was resuspended in 2% SDS and sonicated four times for 10 seconds at an amplitude of 75% and one final time for 10 seconds at 85% amplitude. Samples were then centrifuged at 13.4 k rpm for 20 minutes at 4°C and the supernatant was aliquoted for western blot (WB) and stored at -80°C until needed.

### Primary mouse astrocyte culture and migration assay

Primary astrocytes were prepared from postnatal day 0 or 1 (P0/1) C57BL/6 mice. The cortical tissue was placed in a petri dish already containing a few drops of pre-warmed DMEM/F12 (10-090-CV, Corning) supplemented with 10% heat-inactivated foetal bovine serum (FBS) and 1% penicillin/streptomycin (p/s) (complete media). The tissue was cross-chopped with a scalpel and placed in a 15 ml falcon tube, also containing pre-warmed complete media. The tissue was then placed in the incubator for 15 minutes at 37°C. The tissue was gently triturated and the solution was passed through a 100 μm cell strainer (431752, Corning) before being gently centrifuged. The resulting pellet was re-suspended in complete media and plated in T75 flasks, one brain per flask. Cells were grown in the incubator at 37°C and 5% CO_2_ for 12–14 days and the media changed every 2–3 days. The grown cells were vigorously shaken for 2 hours to remove any contaminating microglial cells, trypsinized, resuspended in complete media and plated for experiments. Cells were used at passages 1 or 2. For migration experiments, astrocytes were serum-starved for 2 hours in T75 flasks, washed once with PBS and incubated with 0.25% trypsin for 10 minutes at 37°C. The media and free-floating astrocytes were then centrifuged, and the pellet was resuspended in DMEM/F12 without FBS or p/s (serum-free media (SFM)) to get a suspension of 2 million cells/ml cell. The cells were plated in the top chamber of a transwell assay insert with an 8.0 µm transparent PET membrane (353097, Falcon). The top chamber of the insert contained 100 ng/ml LPS in SFM media (L4391, Sigma) with or without 1 μM clotrimazole (C6019, Sigma) and/or 10 μM NIBR189. Astrocytes were plated in SFM in the top chamber of the insert for 16–18 hours at 37°C, 5% CO_2_ and left to migrate to the bottom chamber. After 16–18 hours, the media inside the insert was discarded and the cells that did not migrate were carefully removed with a cotton swab. Afterwards, the inserts were incubated in crystal violet solution (V5265, Sigma) for 10 minutes at room temperature (RT). Then, they were dipped carefully twice in a beaker with distilled water to remove the excess crystal violet and left to air dry. When dried, images of the inserts were taken with a light microscope at 20 x magnification. Finally, inserts were put in methanol (621990110, Poch) for 10 minutes on a shaker to extract the cells. The obtained solution was transferred to a 96-well plate and absorbance was read at 570/10 nm. Astrocytes which were plated in serum-free media only, without LPS or clotrimazole, were used as control and the other conditions were normalised to the absorbance from the control cells. Values are expressed as % of the migrated cells compared to the non-treated astrocytes (SFM), were 100% represents the entirety of the cells that migrated in the absence of any compound.

### Human cell culture

Gibco human astrocytes (cat. nr. K1884, Lot nr. 802268, Invitrogen) were sourced from a 20 weeks old male donor. The primary human brain microvascular endothelial cells (cat. nr. ACBRI 376, Lot nr. RI-376, Cell Systems) were sourced from a 24 years old female donor. Human brain vascular pericytes (cat. Nr. 1200, Lot nr. 10732, ScienCell) were sourced from a male donor. Conditionally immortalized human astrocytes clone 35 (HASTR/ci35), human brain vascular pericytes clone 37 (HBPC/ci37) and human brain microvascular endothelial cells (HBMECs) clone 18 (HBMEC/ci18) were then immortalised by professor Tomomi Furihata. HASTRs, HBPCs and HBMECs were cultured according to established protocols [27]. In brief, HASTRs were cultured in DMEM (10-013-CV, Corning) supplemented with 10% FBS, 1% N2 supplement-A (07152, Stemcell) and 1% P/S [28]. HBPCs were cultured in pericyte media: 2% FBS, 1% pericyte growth supplement and 1% P/S (1201, ScienCell) [29]. EBM-2 BM (Lonza, CC-3162) was used to culture HBMECs, without gentamicin, supplemented with 10 mM GlutaMax and 1% P/S [28]. All cells were maintained at 33°C, 5% CO_2_ during proliferation, with 4 µg/ml of blasticidin S in the media to keep them immortalized. Cells where then moved to 37°C, 5% CO_2_, without blasticidin S, for differentiation before the experiments.

Cells were plated separately on collagen I coated 6-well plates (7.10^5 cells/cm2), two wells per condition. After 48 h in complete media, HBPCs and HBMECs were starved in SFM and treated with 100 ng/ml LPS (L4391, Sigma) for 4, 18 or 24 hours. Each timepoint had its respected untreated control, SFM. After the treatment, cells were washed in PBS and collected in fenozol for real-time quantitative polymerase chain reaction (RT-qPCR).

### Mouse microvessel isolation

P10 C57BL/6 mice were used for brain microvessel isolation according to the protocols by Paraiso et al. and Hartz et al. [30,31]. Briefly, the brain was extracted from the skull, the cerebral cortex was separated from the rest of the brain and the meninges were removed. Cortices were placed in the isolation buffer: 5 mM glutaMAX and 1 mM sodium pyruvate in DPBS with calcium and magnesium, pH 7.4 at 4^o^C. Cortices were then homogenised in the isolation buffer with a Dounce homogeniser for 20 strokes. The homogenate was moved to 2 ml tubes and then 40% polysucrose 400 (P7798-100 g, Merck) in DPBS with calcium and magnesium was added in equal volumes for a final concentration of 20%. After vigorous shaking, the homogenates were centrifuged at 7.7 k rpm for 15–20 minutes at 4°C (fixed angle rotor). The pellet was resuspended in 1% bovine serum albumin (BSA) in the isolation buffer and was then filtered through a 300 µm mesh. The mesh was washed with 4 ml of the BSA-containing isolation buffer. The flow-through was filtered through a 30 µm cell strainer twice and the mesh was washed twice with BSA-containing isolation buffer before discarding the filtrate. The filter was turned upside-down and washed twice with BSA-isolation buffer to wash off the capillaries. After collection, the capillaries were centrifuged at 1,500 rpm for 3 minutes at 4°C and resuspended in isolation buffer or fenozol for subsequent analysis.

### IHC of isolated brain microvessels

After isolation, mouse capillaries were plated in PBS on poly-D-lysine coated 8-well µ -slide plates (80826, Ibidi) and left to adhere for 2 hours in the incubator at 37°C and 5% CO_2_. The media was removed and the capillaries were left to air dry for 30 minutes. The capillaries were washed once in PBS and then fixed in 4% PFA for 10 minutes. The PFA was removed and 50 µl of ice-cold methanol was added for 1 minute. The capillaries were then washed twice with PBS at RT and blocked for 1 hour in 0.5% NGS, 1% BSA and 0.1% Tween20 in PBS. Subsequently, the capillaries were incubated overnight at 4°C in 0.5% BSA and 0.05% Tween20 in PBS with the primary antibodies. Primary antibodies used for mouse microvessels (1:100) were: goat polyclonal EBI2 (RRID: AB_10903697, ab121001, Abcam), rabbit polyclonal CH25H (600-401-MM8, ThermoFisher), mouse monoclonal CYP7B1 (OTI1G7) (TA807549, ThermoFisher), rabbit polyclonal HSD3B7 (RRID: AB_10856786, BS-2366R, ThermoFisher), rabbit monoclonal GFAP (RRID: AB_2631098, 12389, CellSignalling), mouse monoclonal GFAP antibody (1:200) (RRID: AB_2827276, SAB5201104, Sigma-Aldrich), mouse monoclonal CD31 (PECAM-1) (RRID: AB_10596359, BMS137, eBioscience), rabbit polyclonal CD31 (PECAM-1) (RRID: AB_10981955, PA5-16301, ThermoFisher), goat polyclonal PDGFRβ (RRID: AB_2162633, AF1042, R&D Systems), rabbit monoclonal PDGFRβ (RRID: AB_10985851, MA5-15143, ThermoFisher). The capillaries were washed twice with PBS, once in 0.5% BSA with 0.05% Tween20 in PBS for 10 minutes and incubated with secondary antibodies and Hoechst33342 (H1399, ThermoFisher) for 1 hour at RT in the dark. The following secondary antibodies were used: goat anti-mouse IgG (H + L) cross-adsorbed Alexa Fluor 546 (RRID: AB_2534071, A-11003, ThermoFisher), goat anti-mouse IgG (H + L) cross-adsorbed Alexa Fluor 488 (RRID: AB_2338840, 115-545-003-20, JacksonImmuno), goat anti-rabbit IgG (H + L) cross-adsorbed Alexa Fluor 488 (RRID: AB_2630356, ab150077, Abcam), donkey anti-goat IgG (H + L) cross-adsorbed Alexa Fluor 546 (RRID: AB_2534103, A11056, ThermoFisher) and Hoechst33342 (H1399, ThermoFisher). The capillaries were then washed three times in PBS and once in ddH_2_O for 10 minutes, air-dried, mounted with Keiser’s gelatine and imaged with Zeiss LSM880 confocal microscope.

### IHC of mouse brain sections

The mouse sections in anti-freeze were washed in PBS for 3 x 5 minutes and then incubated in cold 4% PFA for 5 minutes at RT followed by incubation in 20% ice-cold methanol for 1 minute. The sections were then washed for 3 x 5 minutes with PBS and blocked for 6 hours at RT with PBS supplemented with 10% BSA, 0.5% Triton-X and 1% normal goat serum (NGS). Blocked sections were incubated overnight at RT with primary antibodies diluted in the following solution: PBS supplemented with 2% BSA and 0.1% Triton-X. Primary antibodies (1:100 unless stated otherwise) used were: goat anti-EBI2 (RRID: AB_1090369, ab121001, Abcam), rabbit polyclonal CH25H (600-401-MM8, ThermoFisher), mouse monoclonal CYP7B1 (OTI1G7) (TA807549, ThermoFisher), rabbit polyclonal HSD3B7 (RRID: AB_10856786, BS-2366R, ThermoFisher), mouse monoclonal Iba1 (GT10312) (1:200) (RRID: AB_2735228, MA5-27726, ThermoFisher), rabbit Iba1 (RRID:AB_839504, 019-19741, Wako), mouse monoclonal GFAP antibody (1:200) (RRID: AB_2827276, SAB5201104, Sigma-Aldrich), rabbit monoclonal GFAP (1:200) (RRID: AB_2631098, 12389, CellSignalling), mouse monoclonal CD31 (PECAM-1) (RRID: AB_10596359, BMS137, eBioscience), rabbit polyclonal CD31 (PECAM-1) (RRID: AB_10981955, PA5-16301, ThermoFisher), rabbit monoclonal PDGFRβ (RRID: AB_10985851, MA5-15143, ThermoFisher), goat polyclonal PDGFRβ (RRID: AB_2162633, AF1042, R&D Systems), rabbit polyclonal occludin (RRID: AB_2533468, 40-4700, ThermoFisher), mouse monoclonal N-cadherin (3B9) (RRID: AB_2313779, 333900, ThermoFisher). The following day, sections were washed 3 x 10 minutes in PBS and incubated for 2 hours at RT with the following secondary antibodies (1:500): goat anti-mouse IgG (H + L) cross-adsorbed Alexa Fluor 546 (RRID: AB_2534071, A-11003, ThermoFisher), goat anti-mouse IgG (H + L) cross-adsorbed Alexa Fluor 488 (RRID: AB_2338840, 115-545-003-20, JacksonImmuno), goat anti-rabbit IgG (H + L) cross-adsorbed Alexa Fluor 488 (RRID: AB_2630356, ab150077, Abcam), donkey anti-goat IgG (H + L) cross-adsorbed Alexa Fluor 546 (RRID: AB_2534103, A11056, ThermoFisher) and Hoechst33342 (H1399, ThermoFisher). The sections were then washed 3 x 10 minutes in PBS, air-dried, mounted with Keiser’s gelatine and imaged using a confocal Zeiss LSM880 microscope.

### Immunocytochemistry (ICC) of human cells

HASTR, HBPC and HBMEC were plated on collagen I (cc076, Merck) coated µ -Slide 8 Well (7.10^5 cells/cm2). After 48 h in complete media, cells were washed in PBS and then fixed for 15 minutes in 4% PFA. Afterwards, cells were washed twice in PBS, then blocked in 2% BSA, 0.5% Triton-X and 1% NGS for 1 hour. Antibody solution consisted of 1% BSA and 0.1% Triton-X. Primary antibodies diluted 1:100 were: goat polyclonal EBI2 (RRID: AB_10903697, ab121001, Abcam), rabbit polyclonal CH25H (600-401-MM8, ThermoFisher), mouse monoclonal CYP7B1 (OTI1G7) (TA807549, ThermoFisher), rabbit polyclonal HSD3B7 (RRID: AB_10856786, BS-2366R, ThermoFisher). Cells were then washed in PBS and incubated with secondary antibodies and Hoechst33342 (H1399, ThermoFisher) in antibody solution for 1 hour. Secondary antibodies diluted 1:500 were: goat anti-mouse IgG (H + L) cross-adsorbed Alexa Fluor 488 (RRID: 183 AB_2338840, 115-545-003-20, JacksonImmuno), goat anti-rabbit IgG (H + L) cross-adsorbed Alexa Fluor 488 184 (RRID: AB_2630356, ab150077, Abcam), donkey anti-goat IgG (H + L) cross-adsorbed Alexa Fluor 546 (RRID: 185 AB_2534103, A11056, ThermoFisher) and Hoechst33342 for 1 hour at RT in the dark. Finally, cells were washed three times in PBS and once in ddH_2_O for 10 minutes, air-dried, mounted with Keiser’s gelatine and imaged with Zeiss LSM880 confocal microscope.

### RT-qPCR of cells and tissue

RNA, from samples in fenozol, was isolated using the total RNA mini plus kit (036-100, AA Biotech) according to the manufacturer’s protocol. In brief, fenozol samples were heated up for 5 minutes at 50°C, 150 µl of water was added to each tube before vertexing. After 5 minutes incubation, samples were spun for 15 minutes at 1.2x10^4 rpm at RT. Subsequently, 400 µl of supernatant was collected and mixed with 400 µl of isopropanol. Then, 800 µl of the mixture was added to the column and spun for 60 seconds at 1.2x10^4 rpm. The eluted part was discarded and 700 µl of wash buffer was added to the column before another 60 seconds spin. The was step was repeated one more time. A final wash was performed with 300 µl and a 2-minute spin. Finally, columns were placed in new tubes and 50 µl of pure water was added to the column. After 2 minutes of incubation, columns were spun for 1 minute at 1.2x10^4 rpm. The eluted portion contains the total RNA which was quantified using a PerkinElmer VICTOR Nivo plate reader to equalise the samples. Purity was also assessed based on the 260/280 ratio. The cDNA synthesis was performed with a transcriba kit (4000, AA Biotech) using the following programme: 60 minutes at 42°C followed by 5 minutes at 70°C or using the high-capacity cDNA reverse transcription kit (4368814, ThermoFisher) with the following programme: 10 minutes at 25°C followed by 120 minutes at 37°C and 5 seconds at 85°C. The RT-qPCR was then performed with the TaqMan fast advanced master mix (4444557, ThermoFisher) or the Sensitive RT HS-PCR Mix (2017-2000, AA Biotech) on the LightCycler480 (Roche) according to the manufacturer’s protocol. All samples were run in duplicate. The following FAM dye-labelled Taqman (Applied Biosystems) primers were used: β-actin (Mm02619580_g1 and Hs03023943_g1), GAPDH (Mm99999915_g1 and Hs02786624_g1), EBI2 (Mm02620906_s1 and Hs00270639_s1), CH25H (Mm00515486_s1 and Hs02379634_s1), CYP7B1 Mm00484157_m1), HSD3B7 (Mm01159156_g1 and Hs00986913_g1), occludin (Mm00500912_m1), N-cadherin (Mm01162490_m1). The relative gene expression was determined after normalisation to the housekeeping gene using the ΔCt (baseline gene expression in normal tissue) or ΔΔCt for comparisons of gene expression between treated and untreated samples.

### Enzyme-linked immunosorbent assay (ELISA)

ELISAs were performed using DuoSet ELISA kits: mouse IL-6 (DY406-05), mouse IL-1b/1F2 (DY401-05) and mouse TNF-α (DY410-05) from R&D Systems according to the manufacturer’s instructions. Briefly, 100 µl of samples or standards in reagent diluent, 1% BSA in PBS, were added to a 96-well plate and incubated for 2 hours at RT. The wells were then emptied and washed three times with the wash buffer, 0.05% Tween-20 in PBS. Subsequently, 100 µl of the detection antibody diluted in the supplied reagent diluent was added to each well for 2 hours at RT. The plate was washed again with the wash buffer and 100 µl of streptavidin-HRP was added to each well for 20 minutes at RT in the dark. The plate was again washed three times and 100 µl of the substrate solution, 1:1 H_2_O_2_ and tetramethyl benzidine (34021, LifeTechnologies), was added to each well for 20 minutes at RT. To stop the reaction, 50 µl of H_2_SO_4_ 1 mol/L, was added to the wells and mixed gently. Optical density was read at 450 nm with a VICTOR Nivo plate reader (PerkinElmer).

### RNA sequencing

RNA-sequencing was performed by Genewiz, Tokyo, Japan with 200 ng of total RNA from (i) untreated HBMECs, (ii) untreated or TNFα treated HBPCs; (iii) untreated or treated HASTRs with TNFα/IL1α/C1q 50 ng/ml,10 ng/ml, 500 ng/ml respectively, all from PeproTech except for C1q from Calbiochem) or TNFα/IL1β for 96 hours. The NEBNext Ultra II RNA Library Prep Kit for Illumina (New England BioLabs, MA, USA) was performed on the RNA, after quality assessment, for cDNA library construction. Paired-end sequencing of 150 bp was used for RNA sequencing on the Illumina NovaSeq 6000 platform (Illumina, Inc., San Diego, CA, USA). The GRCh38 (hg38) was used to align the readings of the RNA sequence and quantification of gene expression levels was performed using HTSEQ v0.6.1. A different dataset from the same RNA-seq study was previously published: HBMECs [32] and HBPCs [33].

### Data analysis

All statistical analysis was performed with GraphPad Prism 9 applying unpaired student t-tests for comparisons of two groups, one-way analysis of variance (ANOVA) for comparisons of three or more groups followed by Tukey’s multiple comparisons post-hoc tests or one-sample t-test for comparisons of three or more groups to normalised control. Data are shown as mean + /- standard error of the mean (SEM). Where appropriate, p values are written in the figure legends with significant effects indicated by asterisks: * p < 0.05, **p < 0.01, ***p < 0.001, ****p < 0.0001. Grubbs’ tests were run to identify and remove outliers.

## Results

### The oxysterol 7α,25OHC synthesising and degrading enzymes are expressed in the mouse brain microvessels

**Fig 1:**
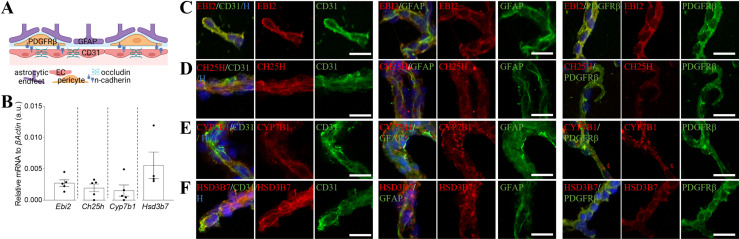
The oxysterol 7α, 25OHC synthesising and degrading enzymes are expressed in the mouse brain microvessels. A. Simplified graphical representation of the BBB showing the localisation of occludin forming tight junctions between ECs, and the localisation of N-cadherin, an adhesion protein between the pericytes and ECs. B. The mRNA expression of *Ebi2* and the 7α,25OHC synthesising (*Ch25h* and *Cyp7b1*) and degrading (*Hsd3b7*) enzymes were assessed in isolated mouse brain microvessels. Data presented as mean + /- SEM, N = 5 independent microvessel isolations of 2–4 brains per isolation. C.-F. EBI2 and the 7α,25OHC synthesising (CH25H, CYP7B1) and degrading (HSD3B7) enzymes (red) are present in isolated mouse brain microvessels. Co-staining of EBI2 and the enzymes with BBB/vascular cell markers: CD31 (endothelial cells, green), GFAP (astrocytes, green) and PDGFRß (pericytes, green). Nuclei (Hoechst, blue). Scale 25 µm.

With the exception of CH25H which was recently shown to be synthesised by mouse CNS ECs [23], the expression of EBI2 and the second 7α,25OHC synthesising enzyme, CYP7B1, and the degrading enzyme, HSD3B7, in the brain microvascular cells has not been investigated. To examine the presence of EBI2 and the remaining enzymes in 7α,25OHC synthetic pathway in the brain vessels, we isolated healthy mouse brain microvessels to analyse both protein and gene expression of the receptor and enzyme levels. The data showed that mRNA of *Ebi2* and the three enzymes were present in the isolated brain microvessels of healthy mice (Fig 1A and B). Similarly at protein level, EBI2 was detected in cells positive for the pericytes marker, PDGFRβ, the EC marker, CD31, and in astrocytic endfeet (GFAP+) (Fig 1C). The CH25H enzyme was present in CD31 positive cells (ECs), GFAP positive cells (astrocytic endfeet) and slightly less in PDGFRβ positive cells (pericytes) (Fig 1D). The second enzyme in the 7α,25OHC synthetic pathway, CYP7B1, was present in CD31 positive cells, GFAP and PDGFRβ positive cells (Fig 1E). The 7α,25OHC degrading enzyme HSD3B7 was highly present in CD31 + cells (ECs) and PDGFRβ+ cells (pericytes) and to a lesser degree in GFAP + cells (astrocytic endfeet) (Fig 1F).

### LPS induces changes in the expression of Ebi2, Ch25h, Cyp7b1 and Hsd3b7 enzymes in the mouse brain

**Fig 2:**
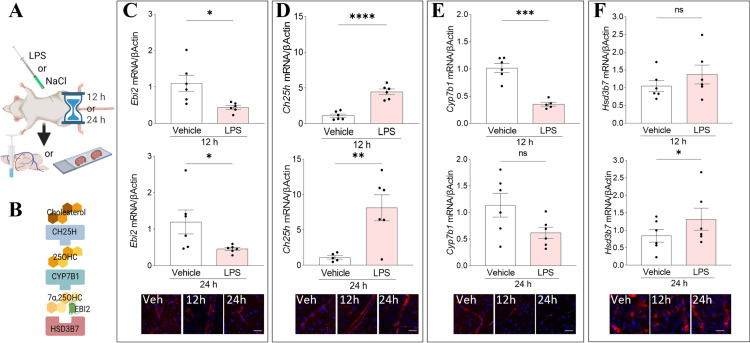
LPS induces changes in the expression of Ebi2, Ch25h, Cyp7b1 and Hsd3b7 enzymes in the mouse brain. A. Schematic representation of experimental setup. B. 7α,25OHC synthesis pathway. C. The mRNA expression of *Ebi2* in whole brain homogenates after 12 and 24 hours of LPS treatment and representative images showing EBI2 (red) in the brain blood vessels after vehicle or LPS administration (12 and 24 h). D. The mRNA expression of the *Ch25h* enzyme in the mouse brain 12 and 24 hours post LPS injection and representative images showing CH25H (red) in the brain blood vessels. E. The mRNA levels of the *Cyp7b1* enzyme 12 and 24 hours after LPS treatment and representative images showing CYP7B1 (red) in the brain blood vessels. F. The expression levels of the 7α,25OHC degrading enzyme, *Hsd3b7*, after 12 and 24 hours of LPS treatment and representative images showing HSD3B7 (red) in the brain blood vessels. Nuclei (Hoechst in blue). Scale 25 µm. Data presented as mean + / − SEM, n = 6 mice, unpaired student t-tests, * p < 0.05, **p < 0.01, ***p < 0.001, ****p < 0.0001 vs. corresponding vehicle.

Several previous *in vitro* studies including one of ours, investigated the effects of LPS on the expression of EBI2 and its ligand’s synthesis pathway on various human and mouse cells [2,5,19,22]. In one study human subjects were injected intravenously with LPS and increased plasma levels of 25OHC were observed [24]. In an animal model, specifically in the EAE, the concentration of the 7α,25OHC ligand in the CNS was sharply elevated as a result of the enhanced release of 7α,25OHC-synthesising enzymes, CH25H and CYP7B1, leading to an increased brain infiltration by peripheral immune cells [8,19]. Here, using a single high-dose LPS injection, we investigated whether the EBI2/7α,25OHC system is affected in the brain during acute systemic inflammation (Fig 2A and B). The *Ebi2* transcripts in whole brain homogenates were downregulated after 12 and 24 hours of LPS injection (Fig 2C). The first 7α,25OHC synthesising enzyme, *Ch25h*, was strongly upregulated at both time points (Fig 2D) and *Cyp7b1* decreased 12 hours after the LPS challenge and then levelled with the vehicle-treated after 24 hours (Fig 2E). The mRNA levels of the degrading enzyme, *Hsd3b7*, slowly increased after LPS treatment with significant changes observed after 24 hours (Fig 2F). The proteins of interest visualised specifically in the brain vessels in vehicle and LPS (12 h or 24 h) treated mice are shown in corresponding panels below the mRNA data.

Because significant differences between human and mouse BBB-forming cells were observed and described before [28], we also examined the expression of the genes of interest in human brain microvascular endothelial cells (HBMECs), human brain vascular pericytes (HBPCs) and human astrocytes (HASTR) upon stimulation with various pro-inflammatory factors *in vitro* (S1 Fig). RNA-seq analysis revealed that at baseline, HBMECs did not express *EBI2, CH25H* nor *CYP7B1* but expressed the 7α,25OHC degrading enzyme *HSD3B7*. The expression of *EBI2* in HBPCs was downregulated after stimulation with the pro-inflammatory cytokine *TNFα* and the expression of the first enzyme in the 7α,25OHC synthetic pathway, *CH25H*, was upregulated after stimulation. The second enzyme in the synthetic pathway, *CYP7B1*, was not expressed at baseline nor after stimulation with TNFα and the expression of the 7α,25OHC degrading enzyme, *HSD3B7*, was slightly upregulated after stimulation. HASTRs expressed *EBI2* and all three enzymes at baseline. Contrary to the downregulated expression observed in pericytes, *EBI2* transcripts were induced after stimulation with pro-inflammatory cytokines. Similarly to HBPCs, the expression of *CH25H* was upregulated in stimulated HASTRs. The expression of *CYP7B1* remained low after stimulation and *HSD3B7* was slightly downregulated after TNFα/IL1α/C1q and TNFα/IL1β stimulation (S1A Fig). EBI2, CH25H, CYP7B1 and HSD3B7 were detected at protein level in the cultured HASTRs (S1B Fig), HSD3B7 was present in HBMECs (S1C Fig) and EBI2, CH25H and HSD3B7 proteins were present in HBPCs (S1D Fig). Rt-qPCR analysis of LPS treated HBMECs and HBPCs (human astrocytes do not express TLR4 receptors [34] and thus were not treated with LPS) revealed no statistically significant differences in *EBI2*, *CH25H* or *HSD3B7* expression.

### Tight junction and adhesion proteins in the brain are downregulated during systemic inflammation

**Fig 3:**
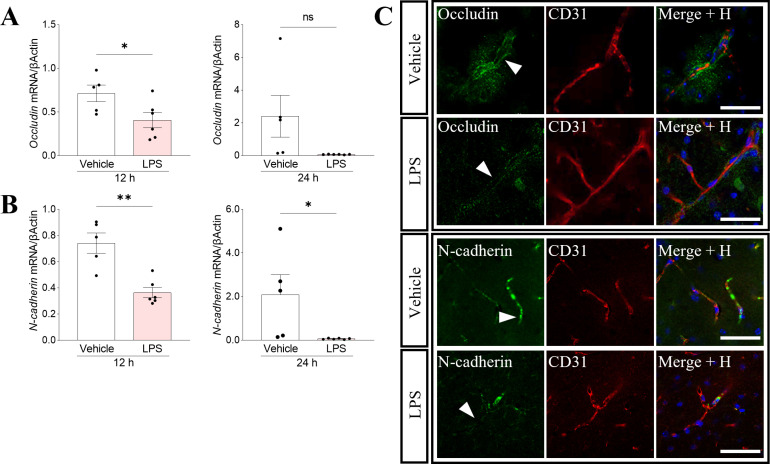
Tight junction and adhesion proteins in the brain are downregulated during systemic inflammation. A. The mRNA levels of *Occludin* were significantly reduced after 12 hours post LPS injection (12 h: 57% + /- 12% vs. vehicle) and started to return to the vehicle levels after 24 hours. B. Expression of the adhesion molecule N-cadherin significantly decreased after 12 hours (12 h: 49% + /- 5% vs. vehicle) and 24 h (3% + /- 1% vs. vehicle) of LPS treatment. Data presented as mean + / − SEM, n = 5-6 mice, unpaired t-test, * p < 0.05, **p < 0.01 vs. corresponding vehicle. C. Representative confocal microscope images show decreased occludin (green) and N-cadherin (green) proteins after LPS treatment in the mouse brain blood vessels (ECs, CD31, red). Immunostaining of cortical blood vessels was performed on mouse brain sections cut in the coronal plane. Nuclei (Hoechst in blue). Scale 50 μm.

Peripheral injections of LPS were shown previously to induce neuroinflammation and disruption of the BBB [35,36]. We also demonstrated that repeated peripheral injections with LPS induce a significant increase in the number of pro-inflammatory cytokines in the mouse brain [10]. The disruption of the BBB function and the extent of neuroinflammation depend heavily on the applied protocol, particularly on the dose and frequency of LPS injections [37]. In our study, we administered a single high dose (2 mg/kg) of LPS via intraperitoneal (i.p.) injection. This protocol effectively induced activation of astrocytes and microglia in the hippocampal region (S2A Fig), along with the release of pro-inflammatory cytokines in the mouse brain. The cytokine release was observed at 12 hours (IL6 and IL1β) and 24 hours (IL1β) following the LPS injection (S2B Fig). After we established that single high dose LPS injection induces neuroinflammation and modulates EBI2 and enzyme levels, we investigated the effects on tight junction and adhesion proteins in the brain as an indirect measure of BBB integrity. The mRNA levels of *Occludin* and *N-cadherin* were downregulated after 12 and 24 hours of LPS treatment at the whole brain level (Fig 3A and B) and were also visualised in the brain blood vessels (Fig 3C).

### The CYP7B1 inhibitor, clotrimazole, and the EBI2 receptor antagonist, NIBR189, attenuate LPS-induced astrocyte migration in vitro

**Fig 4:**
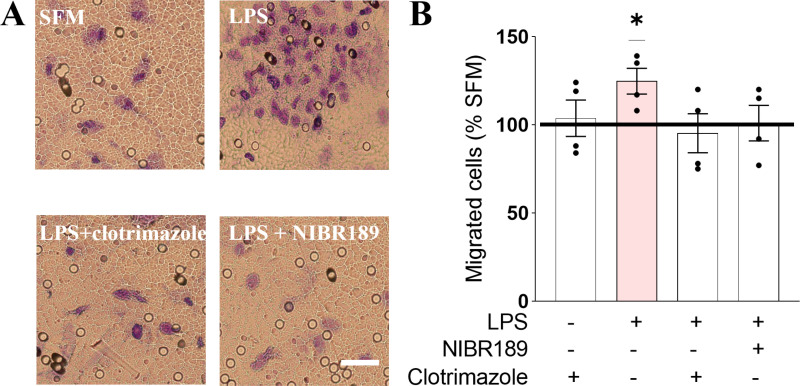
The CYP7B1 inhibitor, clotrimazole, and the EBI2 receptor antagonist, NIBR189, attenuate LPS-induced astrocyte migration *in vitro.* A. Treatment of primary mouse astrocytes with LPS induces chemotaxis. Scale 50 μm. B. Treatment of mouse astrocytes with clotrimazole, a CYP7B1 inhibitor, or NIBR189, a selective EBI2 antagonist, inhibited LPS-induced astrocyte migration (clotrimazole: 76% + /- 9% vs. LPS; NIBR189: 81% + /- 8% vs. LPS). Data presented as mean + / − SEM, n = 4 independent experiments, one-sample t-test t(3) = 3.366, * p < 0.05. SFM: serum-free media.

EBI2 is a chemoattractant receptor shown to induce migration of EBI2-expressing CNS and immune cells *in vitro* and *in vivo* [3–7,9,22]. Macrophages treated with media from LPS-treated astrocytes displayed increased migration, an effect inhibited with the EBI2 antagonist NIBR189, indicating EBI2/oxysterol-dependent mechanisms [2]. *In vivo* studies demonstrated that the trafficking of encephalitogenic CD4 + T cells into the CNS and the severity of EAE are significantly attenuated in CH25H-deficient mice [22]. Increased CNS levels of 7α,25OHC during EAE were found to be the consequence of upregulated synthesis of CH25H by microglia and CYP7B1 by infiltrating lymphocytes and a simultaneous downregulation of HSD3B7. Clotrimazole, a CYP7B1 inhibitor, was shown *in vivo* to downregulate the levels of 7α,25OHC in the mouse spleen leading to altered B cell migration in the follicles, an effect similar to that observed in EBI2 knock-out (KO) mice [16]. Here, we speculated that the concentration of EBI2 ligand increases in blood vessels localized at the site of inflammation and coupled with reduced integrity of the BBB (Fig 3), facilitates migration of peripheral and resident cells to the site of injury. To indirectly test this hypothesis, we conducted *in vitro* experiments utilizing primary mouse astrocytes and we investigated the potential of clotrimazole and the EBI2 antagonist NIBR189 (positive control) to inhibit cellular migration. The data showed increased migration of astrocytes upon LPS treatment and inhibition of LPS-induced chemotaxis upon co-treatment with either clotrimazole or NIBR189, a selective EBI2 antagonist (Fig 4) indicating the involvement of the EBI2/oxysterol pathway in inflammation-induced cellular migration in the brain.

## Discussion

The expression of EBI2 and the enzymes involved in 7α,25OHC synthesis and degradation is dynamically regulated under inflammatory conditions [2,5,8,21,22,26]. Identification of the cellular source of oxysterols in the brain, the magnitude and type of response (up- versus down-regulation), as well as the specificity of the cellular response to a given inflammatory factor, may help in the discovery of new drug targets for the modulation of immune cell trafficking into the CNS during systemic infection, neuroinflammatory or neurodegenerative diseases. Here, we first aimed to determine if the EBI2 receptor, CH25H, CYP7B1 and HSD3B7 enzymes are present in normal mouse brain microvessels. We established that EBI2 is present in ECs, pericytes/smooth muscle cells and astrocytic endfeet. The three enzymes (CH25H, CYP7B1 and HSD3B7) were also detected to a variable degree in each cell type indicating that the microvascular cells contribute to the synthesis, and possibly gradient formation and maintenance, of oxysterol 7α,25OHC in the mouse brain vasculature under normal conditions.

Differences between species need to be taken into consideration when translating findings from animal models to humans. We here confirmed such differences between species in the levels of EBI2 and the three enzymes between mouse and human cells which form the BBB. Using various human and mouse *in vitro* and *in vivo* models we identified differences in *EBI2, CH25H, CYP7B1* and *HSD3B7* expression levels in mouse and human brains as well as the brain microvessels under normal and inflammatory conditions [21]. Table 1 summarizes the data here reported except for the human brain and microvessels data, which is currently under review elsewhere. Briefly, *Ebi2/EBI2, Ch25h/CH25H, Cyp7b1/CYP7B1* and *Hsd3b7/HSD3B7* were all expressed in mouse and human brains and microvessels. HBMECs expressed only *HSD3B7*, HBPCs did not express *CYP7B1* and HASTRs expressed all four genes. Inflammatory stimuli, LPS or cytokines (TNFα or TNFα/IL1α/C1q or TNFα/IL1β) differentially modulated the expression levels of these genes in the mouse brain and human cells.

**Table 1:** The mRNA expression levels of *EBI2, CH25H*, *CYP7B1* and *HSD3B7* in mouse and human brains, isolated brain microvessels and human vascular cells under normal and inflammatory conditions.

	Brain		Brain microvessels		Brain vascular cells		Inflammatory response			
Ms	Hu	Ms	Hu	Hu cells		Ms brain	Hu cells		
						LPS	LPS		Cytokines
***Ebi2/EBI2***	√	√	√	√	HBMEC	x	↓	HBMEC	n/a	n/a
					HBPC	√		HBPC	=	↓
					HASTR	√		HASTR	n/a	↑
***Ch25h/ CH25H***	√	√	√	√	HBMEC	x	↑	HBMEC	n/a	n/a
					HBPC	√		HBPC	=	↑
					HASTR	√		HASTR	n/a	↑
***Cyp7b1/CYP7B1***	√	√	√	√	HBMEC	x	↓	HBMEC	n/a	n/a
					HBPC	x		HBPC	n/a	n/a
					HASTR	√		HASTR	n/a	=
***Hsd3b7/HSD3B7***	√	√	√	√	HBMEC	√	↑	HBMEC	=	n/a
					HBPC	√		HBPC	=	=
					HASTR	√		HASTR	n/a	=

Ms: mouse; Hu: human; √ : expressed; x: not expressed; ↑ : upregulated; ↓ : downregulated; = : levels unchanged; n/a: not assessed

We also determined if EBI2, CH25H, CYP7B1 and HSD3B7 are modulated in the brain under inflammatory conditions. Indeed, EBI2 and the 7α,25OHC synthesising enzymes, CH25H and CYP7B1, were highly modulated in the brain during acute LPS-induced inflammation. The degree of modulation differed depending on the enzyme indicating a microenvironment where levels of oxysterols are locally modulated and controlled. Such tightly controlled oxysterol microenvironment is also established and maintained in T cell follicles by differential CH25H, CYP7B1 and HSD3B7 enzyme expression by lymphoid stromal cells and follicular dendritic cells, which locally increase or decrease 7α,25OHC levels, respectively [38]. The established and maintained 7α,25OHC gradient is crucial for the appropriate localization of B cells in the lymphoid tissue and the launching of an immune response. Given that previous research demonstrated that microglia upregulate CH25H in response to LPS [39–41], we speculate that microglia were likely the main source of the increased CH25H transcripts observed in the whole-brain homogenates after LPS treatment in our study.

The mRNA levels of *occludin* and *N-cadherin* were downregulated at the whole brain level after LPS challenge indicating that peripherally-induced inflammation affects the BBB potentially facilitating the entry of immune cells into the brain during inflammation. Moreover, the data revealed increased pro-inflammatory cytokine levels in the brain and astrocyte and microglia activation after LPS demonstrating that peripherally-induced inflammation instigates neuroinflammatory processes involving the resident CNS immunocompetent cells. These observations are in agreement with previous studies showing increased BBB disruption and pro-inflammatory signalling in the CNS after the LPS challenge [41–43].

Interestingly, endogenous downregulation of EBI2 during mycobacterium tuberculosis infection in macrophages was shown to contain early bacterial infection and intracellular survival [44]. The downregulated *Ebi2* expression in the whole brain after the LPS challenge reported here may indicate a protective mechanism during bacterial infection (LPS challenge) in the CNS involving the EBI2 receptor. Similarly, the expression of *Cyp7b1* initially decreased 12 hours after LPS treatment and started to return to baseline after 24 hours at the whole brain level. Whether the mRNA levels of *Cyp7b1* continued to increase after 24 hours of LPS treatment remains to be elucidated. In a previous study of ours, we reported maximal mRNA expression of *Cyp7b1* after 24 hours of *in vitro* treatment with LPS while the levels of oxysterols 25OHC and 7α,25OHC peaked already after 15 hours indicating different dynamics of mRNA levels and oxysterol synthesis [2]. Moreover, Mutemberezi and colleagues [21] demonstrated that the LPS challenge induces 7α,25OHC levels in the mouse brain after as little as 4 hours and declines thereafter.

The 7α,25OHC degrading enzyme, HSD3B7, was present in all three brain vascular cell types here studied and was least affected by inflammatory signalling, as was demonstrated by us before *in vitro* [2]. The RNA-seq of HBMECs, HBPCs and HASTRs also demonstrated that the HSD3B7 enzyme is not affected by pro-inflammatory signalling. It is possible that during inflammation, the 7α,25OHC-synthesising enzymes, CH25H and CYP7B1, are upregulated and the degrading enzyme, HSD3B7, remains constant or is downregulated thus allowing for an increase in the concentration of 7α,25OHC in the inflamed tissue and a subsequent increase in immune cell infiltration of the inflamed tissue.

The concentration of 7α,25OHC in the mouse CNS increases in the EAE model of MS [8,21]. Again, the increase in 7α,25OHC levels results from differential regulation of CH25H, CYP7B1 and HSD3B7 enzymes by different cells including microglia and infiltrating lymphocytes [8]. The subsequent increase in the concentration of the EBI2 ligand, 7α,25OHC, was shown to enhance the migration of autoreactive T cells into the CNS and exacerbate the disease course. Similar results were obtained in the EAE model performed in CH25H knock-out mice where the disease severity was significantly attenuated as a result of reduced trafficking of memory CD4 + T cells into the CNS [22]. Along the same lines, it was recently demonstrated that ablation of CH25H specifically in ECs attenuates EAE [23]. Natalizumab, a disease-modifying therapy for MS, is a humanized IgG4 antibody against the α4-integrin subunit, which works by blocking the interaction between the α4 integrin expressed on lymphocytes and VCAM1 expressed on ECs thus limiting the passage of autoreactive lymphocytes across the vessel walls into the CNS [45]. Similarly, we demonstrated here that increased migration of EBI2-expressing cells, such as CNS resident astrocytes, in response to LPS treatment, is mediated by the EBI2/7α,25OHC system. The increase in chemotaxis was attenuated by inhibition of the ligand’s synthesis (clotrimazole) or directly by blocking the binding of the ligand with EBI2 (antagonist NIBR189). Modulation of the levels of CH25H, CYP7B1 and HSD3B7 enzymes directly in the brain may thus be another way to limit the increased chemotaxis or entry of EBI2-expressing encephalitogenic immune cells into the CNS during acute inflammation, neuroinflammatory disease or neurodegenerative diseases.

## Conclusions

Taken together, these data indicate that the enzymes regulating the levels of 7α,25OHC are expressed directly by the brain microvascular cells. Moreover, the levels of EBI2 and CH25H, CYP7B1 are heavily regulated in the brain during acute peripherally-induced inflammation. Modulation of EBI2 signalling and/or local concentrations of CH25H, CYP7B1 and HSD3B7 in the brain and the brain blood vessels might result in disease-modulatory effects with potential therapeutic applications in the treatment of neuroinflammatory diseases including multiple sclerosis.

## Supporting information

S1 Fig Expression of *EBI2, CH25H, CYP7B1* and *HSD3B7* enzymes in human brain vascular cells. A. Single-cell RNA-seq data showing expression of *EBI2, CH25H, CYP7B1* and *HSD3B7* in human brain microvascular ECs (HBMECs), human brain vascular pericytes (HBPCs) and human brain astrocytes (HASTRs) at baseline (unstimulated/control cells) and after stimulation with TNFα (HBPCs), TNFα/IL1α/C1q or TNFα/IL1β (HASTRs). Gene expression unit is: fragments per kilobase of transcript per million mapped fragments (FPKM). B. IHC staining shows EBI2, CH25H, CYP7B and HSD3B7 (all in green) in cultured HASTRs. Nuclei (Hoechst in blue). Scale 100 µm. C. IHC staining shows HSD3B7 (green) in cultured unstimulated HBMECs, nuclei (Hoechst in blue), scale 100 µm. There were no statistically significant differences in HSD3B7 mRNA expressionn after stimulation with 100 ng/ml LPS, N = 3 independent experiments. The red dotted line indicates expression in untreated cells. D. EBI2, CH25H and HSD3B7 (all in green) are present in cultured HBPCs. Nuclei (Hoechst in blue). Scale 100 µm. There were no statistically significant differences in *EBI2, CH25H* and *HSD3B7* upon stimulation with 100 ng/ml LPS. N = 3 independent experiments, the red dotted line indicates expression in untreated cells to which each experiment was normalised
(TIF)

S2 Fig I.p. injection of LPS induces inflammation in the brain A. I.p. injection of LPS induces mild neuroinflammation as indicated by increased astrocyte (GFAP, red) and microglia (Iba1, red) reactivity. Representative images, scale 50 μm. Nuclei (Hoechst in blue). Immunostaining was performed on mouse brain sections cut in the coronal plane. Images show GFAP and Iba-positive cells in the hippocampal region. B. The levels of pro-inflammatory cytokines in the whole brain homogenates increased after 12 h (IL6: 227% + /- 23% vs. vehicle; IL1β: 416% + /- 53% vs. vehicle) and 24 h (IL1β: 189% + /- 27% vs. vehicle). TNFα levels did not change after LPS treatment. Data presented as mean + / − SEM, n = 6 mice, unpaired t-test, **p < 0.01; ***p < 0.001 vs. corresponding vehicle.
(TIF)
