# Supplementary figures and images for: Differential expression and modulation of EBI2 and 7α,25-OHC synthesizing (CH25H, CYP7B1) and degrading (HSD3B7) enzymes in mouse and human brain vascular cells
Source: PLoS One. 2025 Feb 25;20(2):e0318822. doi: 10.1371/journal.pone.0318822 (PMC11856462; doi:10.1371/journal.pone.0318822)

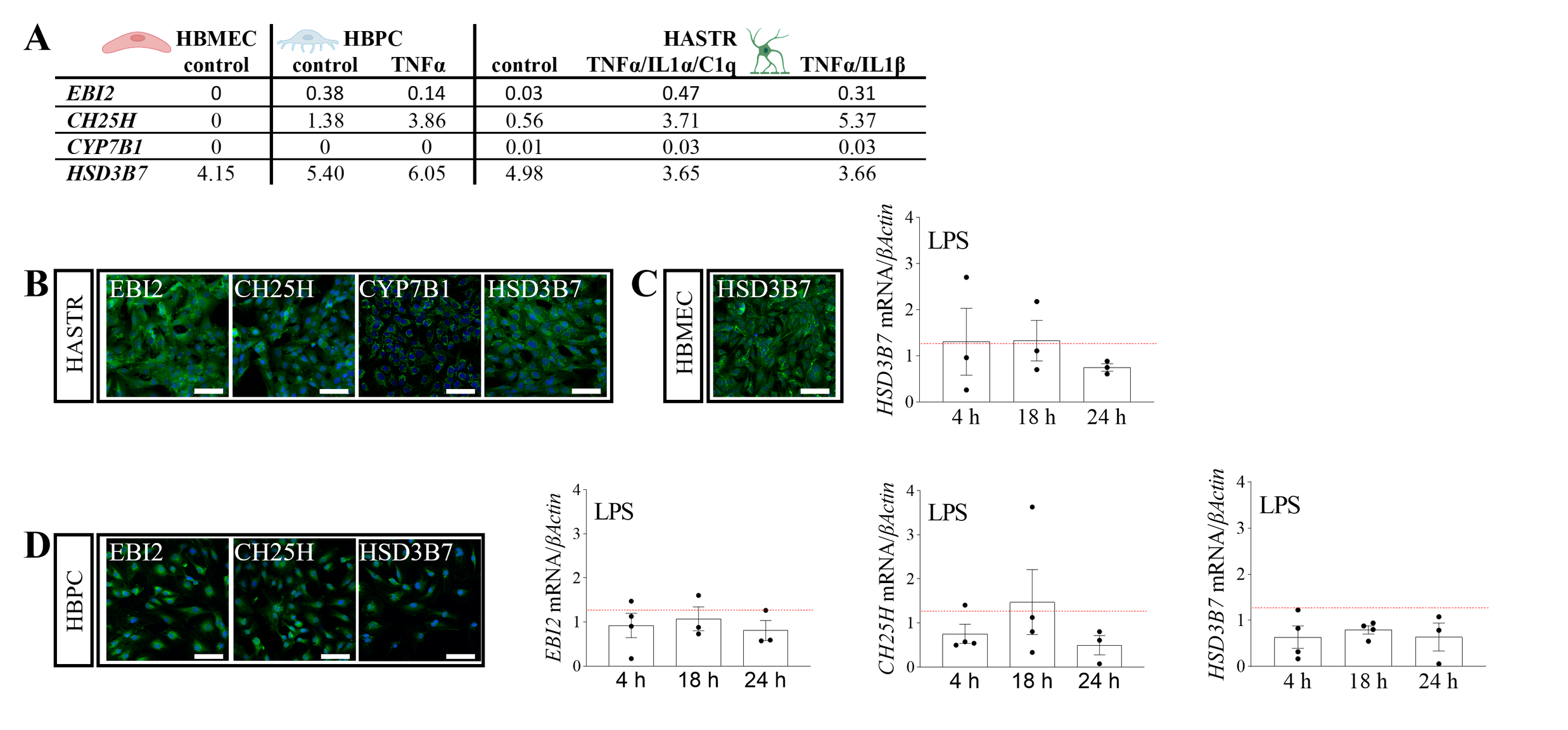

Supplement: S1 Fig — A. Single-cell RNA-seq data showing expression of EBI2, CH25H, CYP7B1 and HSD3B7 in human brain microvascular ECs (HBMECs), human brain vascular pericytes (HBPCs) and human brain astrocytes (HASTRs) at baseline (unstimulated/control cells) and after stimulation with TNFα (HBPCs), TNFα/IL1α/C1q or TNFα/IL1β (HASTRs). Gene expression unit is: fragments per kilobase of transcript per million mapped fragments (FPKM). B. IHC staining shows EBI2, CH25H, CYP7B and HSD3B7 (all in green) in cultured HASTRs. Nuclei (Hoechst in blue). Scale 100 µm. C. IHC staining shows HSD3B7 (green) in cultured unstimulated HBMECs, nuclei (Hoechst in blue), scale 100 µm. There were no statistically significant differences in HSD3B7 mRNA expressionn after stimulation with 100 ng/ml LPS, N = 3 independent experiments. The red dotted line indicates expression in untreated cells. D. EBI2, CH25H and HSD3B7 (all in green) are present in cultured HBPCs. Nuclei (Hoechst in blue). Scale 100 µm. There were no statistically significant differences in EBI2, CH25H and HSD3B7 upon stimulation with 100 ng/ml LPS. N = 3 independent experiments, the red dotted line indicates expression in untreated cells to which each experiment was normalised (TIF) [file pone.0318822.s001.tif]

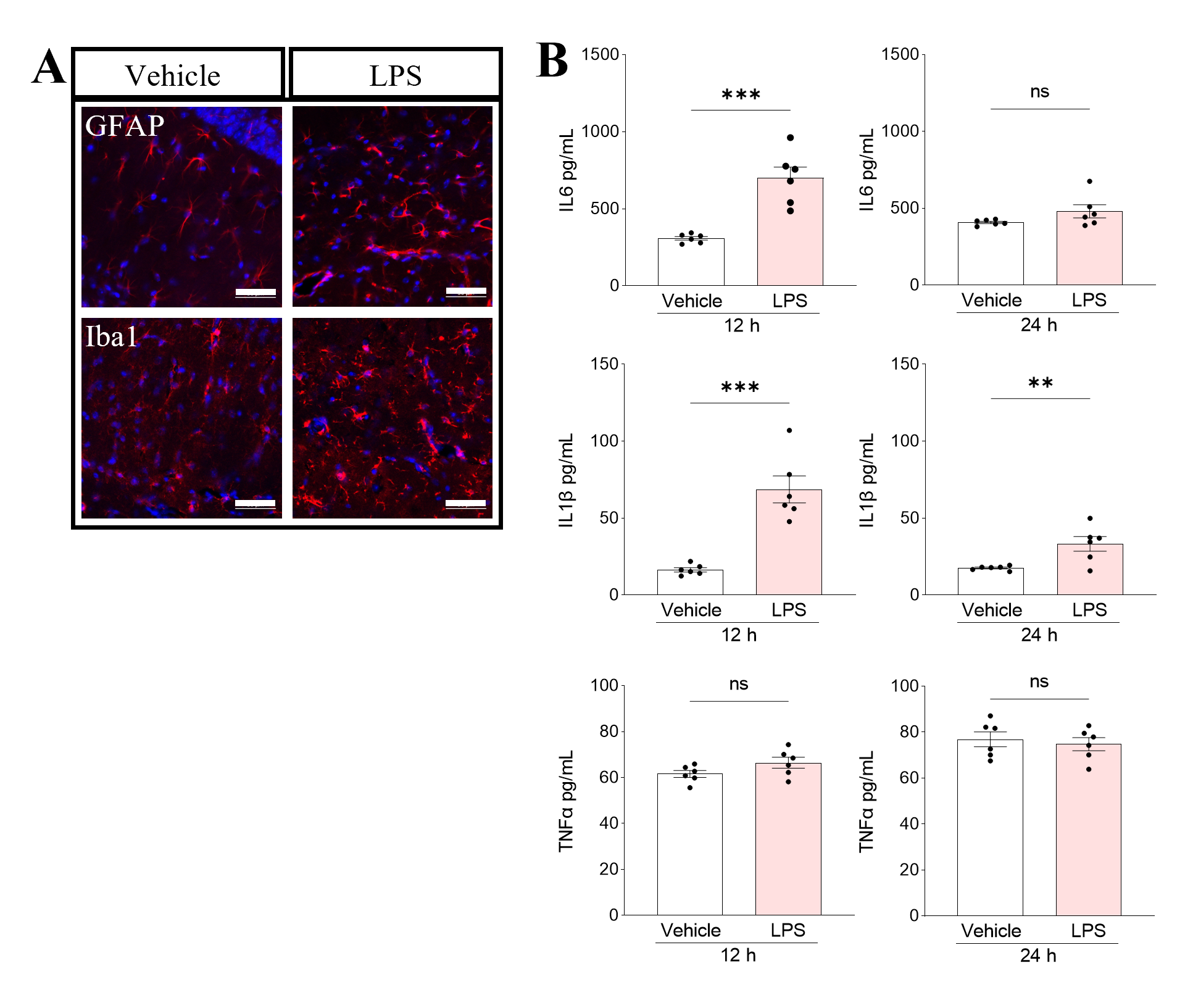

Supplement: S2 Fig — I.p. injection of LPS induces mild neuroinflammation as indicated by increased astrocyte (GFAP, red) and microglia (Iba1, red) reactivity. Representative images, scale 50 μm. Nuclei (Hoechst in blue). Immunostaining was performed on mouse brain sections cut in the coronal plane. Images show GFAP and Iba-positive cells in the hippocampal region. B. The levels of pro-inflammatory cytokines in the whole brain homogenates increased after 12 h (IL6: 227% + /- 23% vs. vehicle; IL1β: 416% + /- 53% vs. vehicle) and 24 h (IL1β: 189% + /- 27% vs. vehicle). TNFα levels did not change after LPS treatment. Data presented as mean + / − SEM, n = 6 mice, unpaired t-test, **p < 0.01; ***p < 0.001 vs. corresponding vehicle. (TIF) [file pone.0318822.s002.tif]
